# Manufacturing and Structural Features with Respect to the Modal Behavior of a Carbon Fiber-Reinforced Epoxy Drum Shell

**DOI:** 10.3390/ma12244069

**Published:** 2019-12-06

**Authors:** Manuel Ibáñez-Arnal, Luis Doménech-Ballester, Fernando Sánchez-López

**Affiliations:** Department of Mathematics, Physics and Technology Sciences, University CEU Cardenal Herrera, CEU Universities, Avda. Seminario s/n, 46113 Moncada, Valencia, Spain; luis.domenech@uchceu.es (L.D.-B.); fernando.sanchez@uchceu.es (F.S.-L.)

**Keywords:** manufacturing, drum, shell, modal, analysis, composite, CFRE

## Abstract

This work evaluates the use of structural aspects in the manufacture of drum shells based on their modal behavior. The drum shells are made of composite carbon fiber-reinforced epoxy (CFRE) due to the structural variables commonly used in the industry for the manufacture of these musical instruments. Musicians consider the shell of a membranophone to be responsible for the differences in timbre between different instruments. Normally, this variation focuses attention on the mechanical characteristics of the material and on the overall thickness of the cylinder that forms the shell. Some manufacturers, especially those that use metals and composites, resort to low thicknesses, below 2 mm, which forces them to use structural reinforcements at the edges of the cylindrical shell to avoid deformations due to the tension generated by the membranes. As shown in this research work, these structural elements have great relevance within the acoustic behavior of the drum shell. Comparisons are made among the frequencies obtained for the different vibrational modes by using finite element simulations, establishing the length of the structural solution previously mentioned and the number of plies of composite laminate as design variables, starting from the characteristics of a real case constructed with CFRE and concluding with experimental validation. The range of study is limited to the values of the frequencies generated by the membranes. The results demonstrate that the use of different manufacturing variables can lead to savings in production costs without compromising the modal behavior of the shell.

## 1. Introduction

Currently, there are many research works about new materials in the manufacturing industry of musical instruments. In recent years, the use of composite materials has become popular. Many manufacturers are opting to replace composites with traditionally used materials such as wood [1,2,3,4], and research is being completed on the acoustic effects that these materials provide [5,6,7,8]. Studies in musical instruments are based on optimizing and designing the resonances of elements, such as plates, soundboards, resonance boxes, and shells, so they can enhance the sound pressure levels of the main vibration elements such as strings, membranes, and air. There is extensive research on the modal behavior of many families of instruments [9,10], such as the cordophones (guitars, pianos, cellos, etc.) [11,12,13,14,15]. For the family of membranophones, numerous studies focus on aspects of membranes such as their modal behavior, radiation patterns [9,16,17,18,19], and how the vibratory behavior of the cylinder conforms to the shell [20,21,22]. The resonance of any solid can be defined as the product of its wave number and its speed of sound. Its wave number *k* is related to the properties of the modal and geometric resonance, while the speed of sound $c_{L}$ is related to the elastic mechanical properties of the material [23,24,25]:

(1) $$f_{r}=\;kc_{L}$$

In the case of the ideal cylindrical membranophone shell, Rossing [9] describes the resonances for the vibrational modes of type (*m*,0) using the following Equation:

(2) $$f_{r}=\;\left(\frac{E}{\rho }\right)\left(\frac{h}{2\pi r^{2}\sqrt{12}}\right)\left[\frac{m\left(m^{2}-1\right)}{\sqrt{m^{2}+1\;}}\right]$$

where *E* is the Young modulus, ρ is the density, *h* is the shell thickness, *r* is the radius of the cylindrical shell, and *m* is the number of axial nodal lines. Equation (2) shows that any change in geometry is likely to lead to changes in the resonances of the cylindrical shell. Research in this field may help manufacturers avoid working with multiple processes and materials and allow them to focus on the manufacture of a single material instead. In addition, geometrical modifications that do not imply a general dimensional change of the product offer the manufacturer the opportunity to modify the modal behavior of the shell of a membranophone at a relatively low cost, as they do not require the modification of molds and tools for production.

The ideal construction parameters for this purpose are the total thickness *h*, generated by the total number of layers of composite laminate, and the structural reinforcements of the shell, which modify the total bending stiffness of the shell, thereby modifying the moment of inertia of the cylinder and Equation (2) and therefore the entire modal behavior of the shell.

In the first case, the thickness increment involves an increase in the use of material, so obtaining certain frequency values in resonances can be expensive.

**Figure 1:**
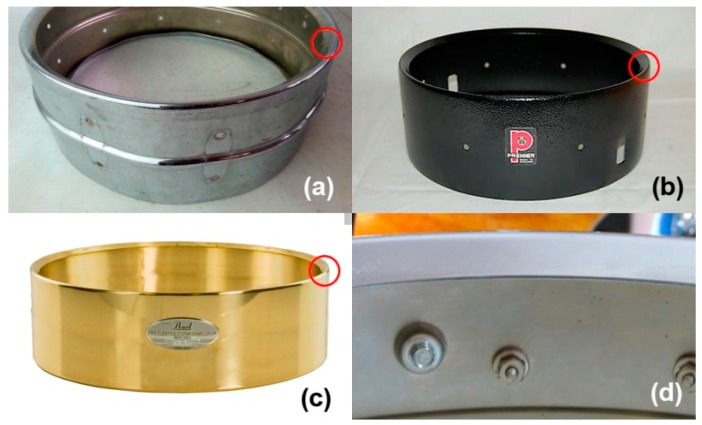
Different drum shells that use structural reinforcements in the form of material folds: (**a**) Ludwig Supraphonic, (**b**) Premier Drum Shell, (**c**) Pearl Free Floating, and (**d**) Slingerland Drum Shell.

In the latter case, as shown in Figure 1, structural reinforcements are used in the form of a fold of material. They support the forces to which an instrument is subjected via the tensing the membranes. They often require only a slight increase in material, so they are a very effective and easily adjustable element for obtaining the desired resonances.

**Figure 2:**
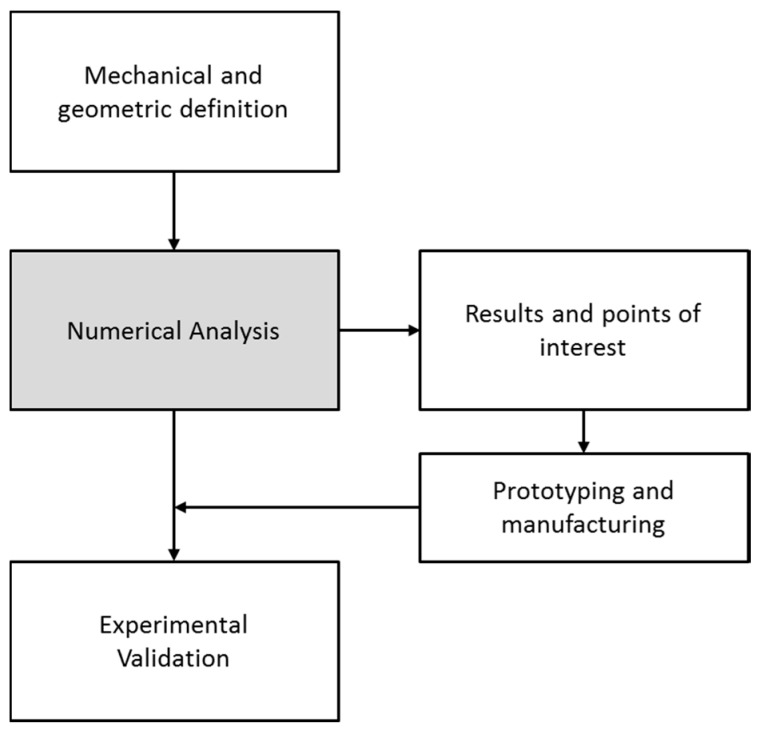
Schematic of the procedures performed in the research.

Understanding how variables influence the structural design can provide a competitive advantage in this industrial sector by reducing the costs associated with the use of materials and the time required for production. For this reason, this paper focuses on the study of possible geometric combinations, considering the thickness and length of the shell’s structural reinforcements. By combining finite element simulations and their experimental validations, it allows us to observe the changes in the modal behavior of the shell and to quantify the differences in frequency values associated with each of the main resonances, as shown in Figure 2.

## 2. Drum Features

### 2.1. Geometrical Aspects

For the purpose of this study, a composite membranophone model constructed in standardized measurements and available on the market is taken as a reference.

**Figure 3:**
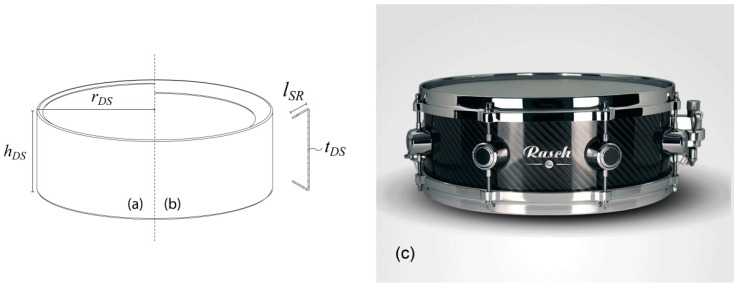
Distribution of geometric variables and comparison of two cases with different *l_SR_*: (**a**) *l_SR_* = 10 mm and (**b**) *l_SR_* = 30 mm. (**c)** Real model used as reference.

It is a snare drum with a cylindrical shell made of carbon fiber-reinforced epoxy (CFRE). The geometric characteristics of the shell of this model are shown in Figure 3. Where some inherited characteristics are taken to determine that the height of the shell $h_{DS}=127\;\mathrm{mm}$ and the radius $r_{DS}=177.8\;\mathrm{mm}$, the length of the structural reinforcement $l_{SR}$ and the number of plies (*N*) (defining the thickness $t_{DS}=N\cdot t_{p}$ being $t_{p}$ the plie thickness) are considered study variables.

### 2.2. CFRE Laminate Characterization and Manufacturing

**Figure 4:**
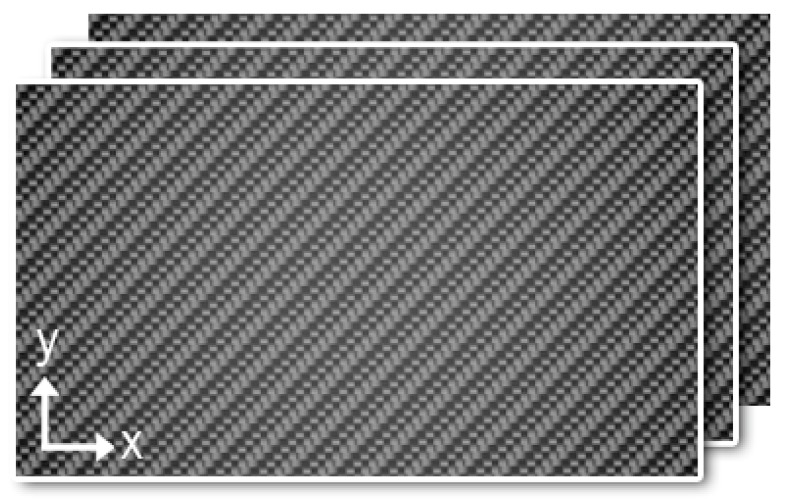
Stacking sequence with collinear plies {0º/0º/0º/...}.

**Figure 5:**
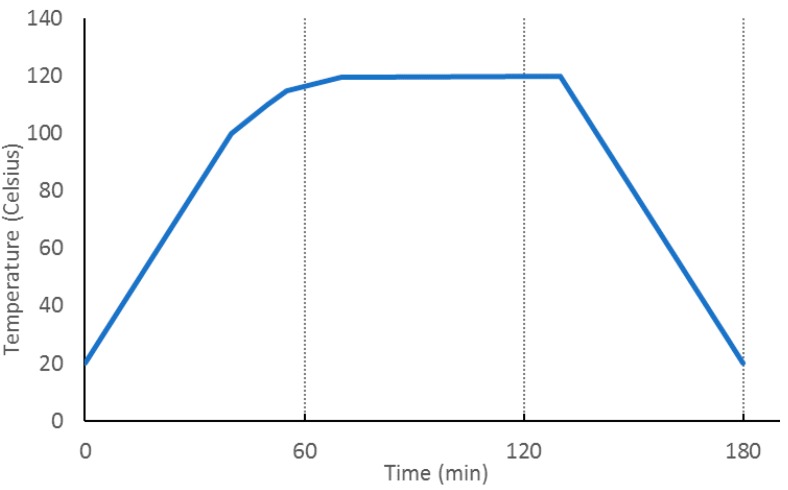
Postcure cycle.

The shell of this reference model consists of a composite laminate resulting from the stacking of three woven layers of carbon fiber epoxy. The multipurpose CFRE system considered for this investigation, shown in Figure 4, was GG280T (Tenax HTA-3k)-DT806R-42 fabric laminate manufactured by the external company, Magma Composites S.L, using prepreg plies supplied by Delta Preg Composites. DT806 is an epoxy resin with a glass transition temperature (Tg) of approximately 135 °C. GG280T is a 4/4 twill carbon fabric with a 3 K high-strength (HS) carbon fiber reinforcement and a density of 198 g/cm^2^. The shell shown in Figure 5 was processed using an autoclave method (pressure 4.05 × 10^5^ Pa) laying three or more collinear plies, as shown in Figure 4.

Autoclave processing allows the elimination of voids generated during the curing process, which eliminates the effects of composite damping [26]. In order to obtain the highest mechanical properties of the composite, a postcure was performed following the curve shown in Figure 5.

**Figure 6:**
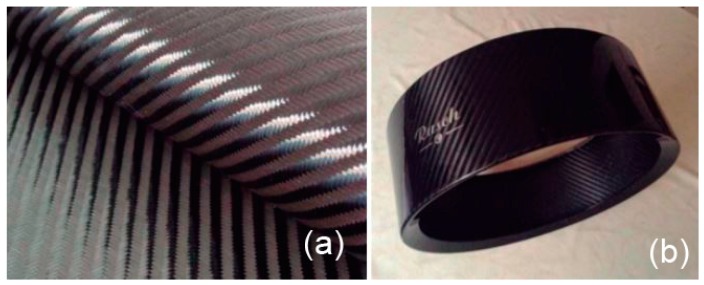
(**a**) Carbon fiber fabric used in the drum and (**b**) drum shell geometry.

The fabric sheets are characterized by two directions of fiber that are perpendicular to each other. In this case, Young’s modulus equivalent is used for both fiber directions $E_{x}=E_{y}$. The resulting thickness plie after processing is 0.34 mm. Elastic material properties used in the construction of this model are shown in Table 1. Material and shell are shown in Figure 6.

### 2.3. Shell Manufacturing

**Figure 7:**
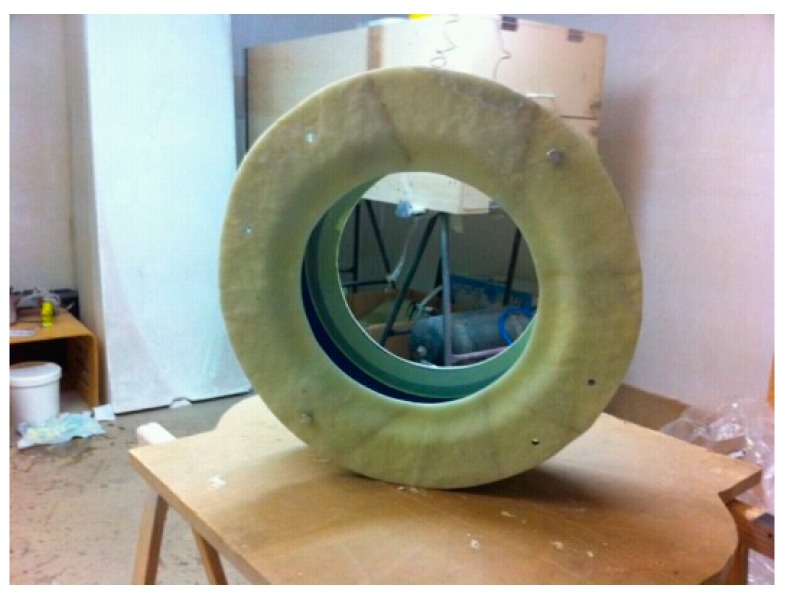
Glass fiber-reinforced epoxy (GFRE) mold developed for the manufacture of the membranophone shell.

To produce the drum shell, a glass fiber-reinforced epoxy (GFRE) mold with an epoxy-based coating finish, shown in Figure 7, was manufactured. The mold was divided into four partitions.

**Figure 8:**
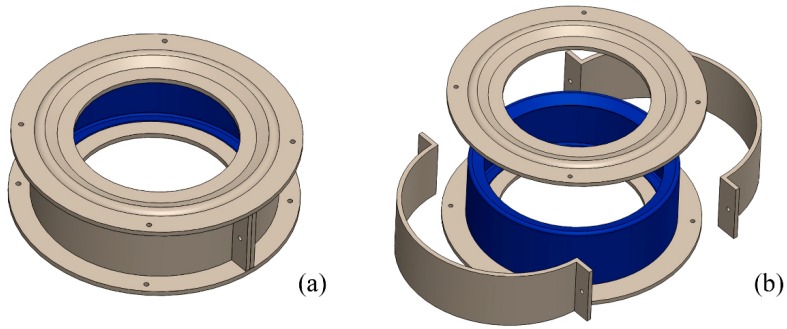
Diagram of assembly and partitions of the mold developed for the manufacture of the CFRE shell: (**a**) assembled mold and (**b**) cured shell demolding.

The upper and lower parts are intended to generate the structural reinforcements described in Figure 8. In addition, two semicircular partitions generate the remaining cylinder that forms the shell.

## 3. Methods

### 3.1. Experimental Methods

One of the simplest and best-known methods for experimental analysis of the different vibrational modes and their direct identification consists of the use of powder placed on an element excited by resonance. This method allows direct observation of the deformations produced due to the accumulation of material in the nodes [27]. There are complex geometries in which the use of this method is impossible, so other methods are required. Holographic laser interferometry allows for observation of the modes of vibration due to the interference of light produced on a vibrating object [27]. Other methods use sensing and data capture on elements excited by impact or resonance, allowing for a detailed analysis of the vibrations and frequencies, which enables identification of the modes of vibration [28,29,30,31].

**Figure 9:**
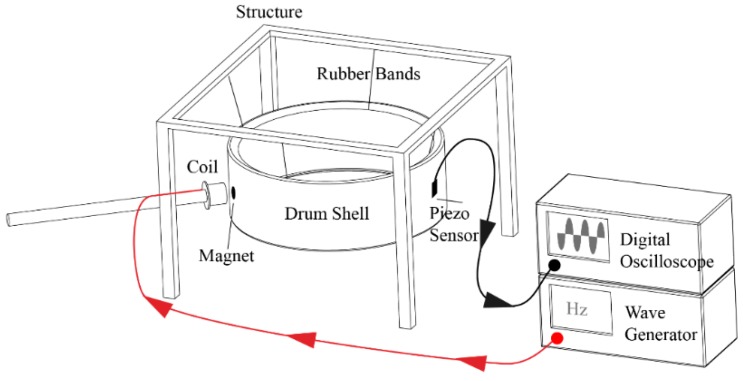
Frequency sweep method performed in the experimental test.

For this research, a resonance detection method based on the external excitation of the shell by sine wave was used. A frequency sweep was performed and the values of each resonance were captured, following the scheme shown in Figure 9.

The wave generator emits a sweep of frequencies that passes to a coil that excites a small magnet attached to the shell. The shell remains in free condition suspended by rubber bands, and piezo-electric sensors capture the displacements generated by the vibrations of the shell. The signals captured by the sensors are analyzed in a digital oscilloscope.

Although each experimental method discussed above has provided good results in previous studies, each requires the construction of one specimen for each study case. The combinations of geometric variables can result in a large number of cases, which is why finite element simulations present great advantages as methodologies in cases where the study to be carried out would involve a significant temporary economic investment. They allow us to carry out a high degree of experimentation without a manufacturing overrun of case studies in order to evaluate their acoustics.

Numerous studies have used the finite element methodology for modal calculation in musical instruments such as the violin and the piano, certifying great agreement with the data obtained experimentally [14,20,32,33,34].

In order to understand how structural reinforcements can influence the vibroacoustic behavior of any drum shell, a set of modal simulations with finite elements was made. Different values of laminate layers were combined with different length values for structural reinforcement. In order to evaluate the vibratory behavior of the drum shell, the values of the frequencies of each mode of vibration were obtained.

### 3.2. Finite Element Analysis

**Figure 10:**
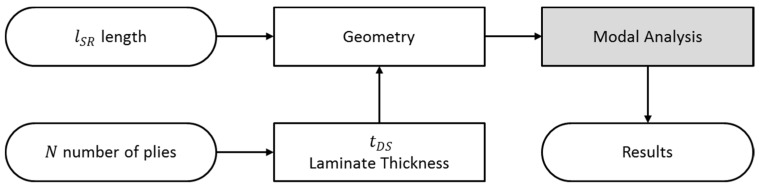
Organizational scheme of the simulations.

The scheme used in the numerical modeling is presented in Figure 10. The used software was ANSYS v17. A parameterization of the study variables ($l_{SR}$ and *N*) was performed. The complete calculation process consisted of the creation of geometry with the structural reinforcement length parameter associated with the study case, which was then sent to the ANSYS Composite Prepost (ACP), where the laminate specified above was generated with the configuration of the composite material with the corresponding number of plies of laminate parameterized for that simulation.

Once the geometry was generated and the total thickness was applied, the vibrational modes and their frequencies were extracted. A list of all the combinations of variables was loaded into the simulation software. All results were stored for further analysis, as will be discussed in the next sections.

### 3.3. Numerical Model

The numerical model used to determine the vibrational modes and their frequencies in an undamped system is

(3) [M]{ü}+[K]{u}={0}

For a linear system, the vibrations must be harmonic with respect to

(4) $$\left\{u\right\}={\left\{\phi \right\}}_{i}cos\omega_{i}t$$

By developing the Equations, we obtained the Equation of the classical problem of eigenvalues, as follows:

(5) $$\left[K\right]{\left\{\phi \right\}}_{i}=\omega_{i}^{2}\left[M\right]{\left\{\phi \right\}}_{i}$$

where  [K] is the stiffness matrix, ${\left\{\phi \right\}}_{i}$ is the mode shape vector (eigenvector) of mode *i*, $\omega_{i}^{2}$ is the eigenvalue, and [M] is the mass matrix [23].

### 3.4. Parameter Study Range

Study limits are established in order to understand the behavior of the frequencies for each mode as a function of the input variables. For the number of plies N, the limits are set to 1<N<9  plies of laminate resulting in a thickness limit of $0.34<t_{DS}<3.06\;$ mm. Although a drum shell of one or two plies is not structurally able to withstand the stresses caused by the drumheads, these have been included in the study to improve the understanding of the overall behavior. The upper limit is established considering that with greater thicknesses, structural solutions would no longer be necessary. The limits for the length of the structural reinforcement are set at 5 < *l_SR_* < 30 mm. In most cases, reinforcements observed in existing products do not exceed 10 mm, even though the study is expanded to confirm the influence of this variable on the resulting frequencies. The upper limit is set at 30 mm, considering that the air must be able to move freely inside the drum shell.

In a membranophone with two membranes, the upper membrane (batter head) is excited by impact. When this happens, vibrations of the membrane are transmitted by direct contact to the shell, which vibrates in a consonant way, transmitting its own resonances back to the membrane. In turn, air contained inside the shell excites the lower membrane. The coupling between the frequencies of both membranes is observed especially in the lower frequencies [35]. Mainly in modes (0,1) and (1,1), pairs of modes resulting from the movements of the membranes in the same or in opposite ways are observed. Since a drum shell can only excite the membrane through different resonances, studies researching this action have focused on the natural frequency range of a two-heads system [17,27,36].

The amplitudes of the vibrations in the membranes decrease rapidly as the mode of vibration (*m*,*n*) increases. Although it normally depends on the point of excitation, generally, the lower frequency modes generate greater amplitudes, especially the fundamental modes (0,1) and (1,1). These modes of vibration present a greater capacity to excite the shell and are therefore considered essential for the possible variations in the timbre.

For a standard tuning, the experimentally observed range for the first nine modes of vibration of the double membrane system is between 182–629 Hz. For this research, considering that in most current membranophones the membrane tension is easily adjustable, the study range was expanded to between 0–2000 Hz in order to cover a wider frequency spectrum. After combining the input variables, a total of 54 different cases were generated for simulation, covering all possible combinations of layers and lengths of structural reinforcements in intervals of 5 mm. Since the evolution curves of the frequencies present a smooth transition, we consider that this distance between points is enough for understanding the evolution of frequencies and optimizing the number of simulations required.

## 4. Results

**Figure 11:**
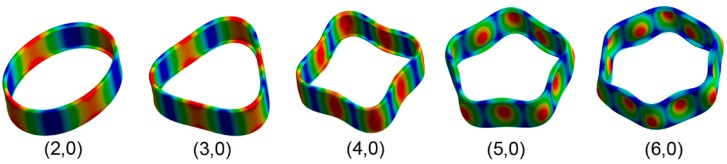
Vibrational modes (m,0) of drum shell obtained from FEM (Finite Elements Modeling) modal simulations.

After performing calculations for the 54 cases, the values of the frequencies and their modal shapes were saved for each mode of vibration. The analysis of the stored data allowed us to understand the frequency behavior due to the variations of the input variables, number of plies of laminate, and length of the structural reinforcement used. For a better understanding and analysis of the obtained data, we opted for a three-dimensional representation that allowed us to visually relate both input variables to their results. In Figure 11, the modes of vibration extracted from the simulations for the range defined for the study can be observed.

**Figure 12:**
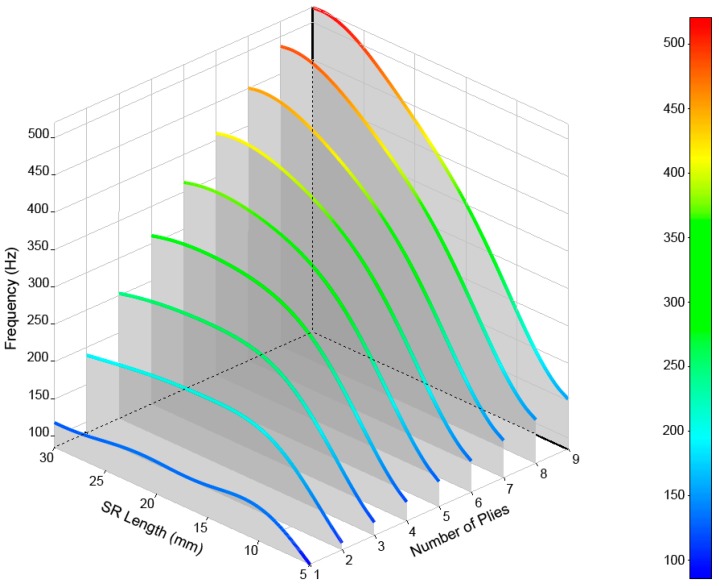
Frequencies obtained for the (2,0) vibrational mode of the drum shell.

**Figure 13:**
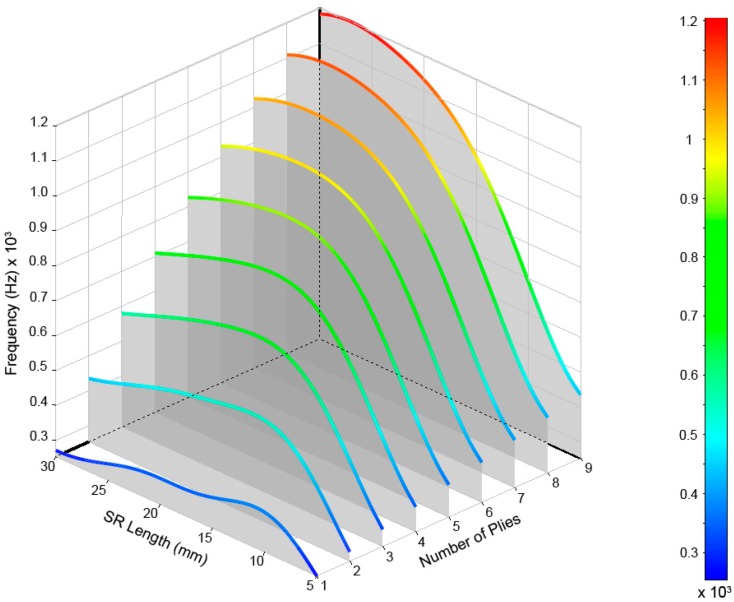
Frequencies obtained for the (3,0) vibrational mode of the drum shell.

**Figure 14:**
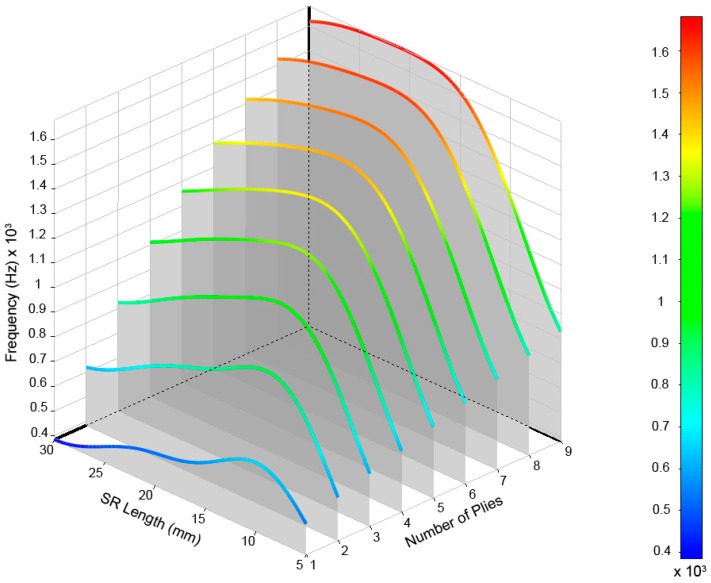
Frequencies obtained for the (4,0) vibrational mode of the drum shell.

**Figure 15:**
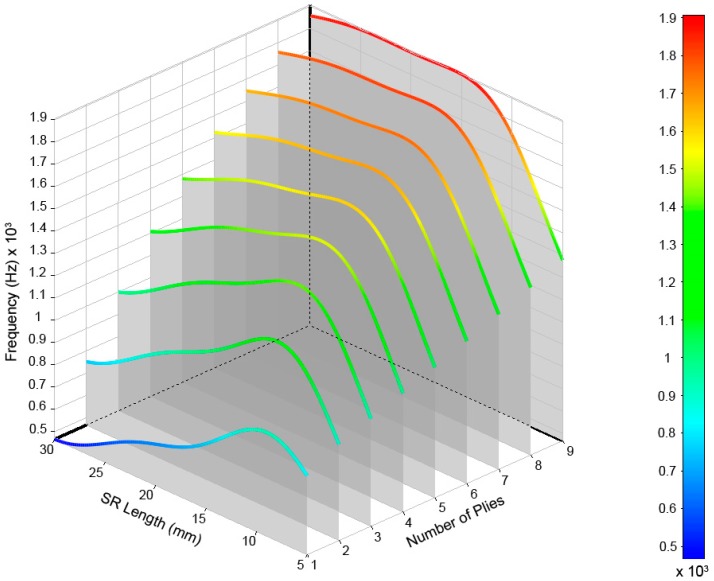
Frequencies obtained for the (5,0) vibrational mode of the drum shell.

**Figure 16:**
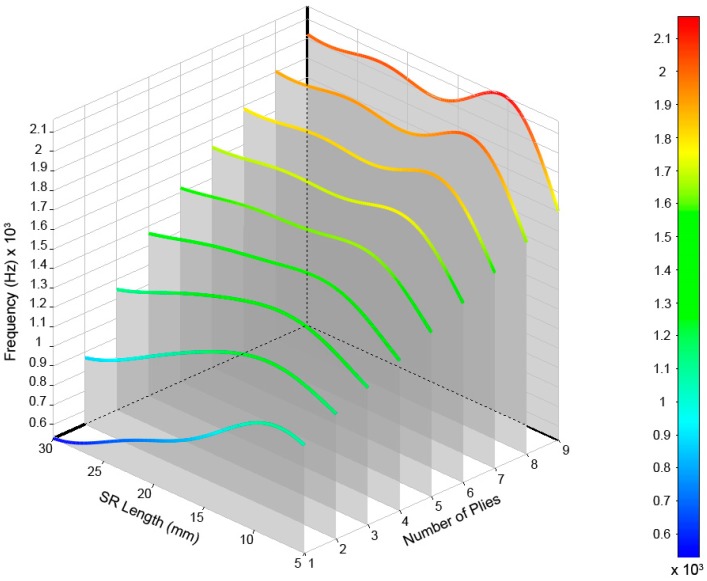
Frequencies obtained for the (6,0) vibrational mode of the drum shell.

The results are shown in Figure 12, Figure 13, Figure 14, Figure 15 and Figure 16 for the different modes of vibration. In each of the graphs, the evolution of the frequencies for a single mode of vibration is analyzed depending on the number of plies and the different lengths of structural reinforcement.

## 5. Discussion

### 5.1. General Effects on the Modal Behavior

In view of the obtained results, we can affirm the sensitivity of the natural frequencies to changes in structural variables of the drum shell. This may represent an acoustic design opportunity, without modifying the general appearance of this type of product.

At a general level, both variables are influential in the modal behavior of the shell. If we analyze the capacity to modify the frequencies of each of the construction variables in Figure 10, Figure 11, Figure 12, Figure 13 and Figure 14, while increases in thickness act by increasing the resonance frequencies for each of the vibration modes, the increase in the length of the structural reinforcement presents maximums from which the frequency is maintained or decreased.

We can also deduce that both variables have a high level of dependency. Increases in the length of structural reinforcement modify the influence of the increase in thickness and vice versa. This makes it essential to study both variables together due to the link between them.

An illustrative example is the one observed for mode (2,0). Its frequency increased by 173% as a result of increasing its lamination in nine layers with an $l_{SR}$ = 5 mm; however, the increase of two layers produces a frequency increase of 214% if we use an $l_{SR}$ = 30 mm. While an increase in frequency occurred in both cases, the combination of both design variables allowed for a 77% savings of material.

### 5.2. Design Optimization Opportunities

Due to the differences in modal behavior generated by the combination of the geometrical variables used in the study, there are different possibilities for obtaining a specific resonance frequency.

**Figure 17:**
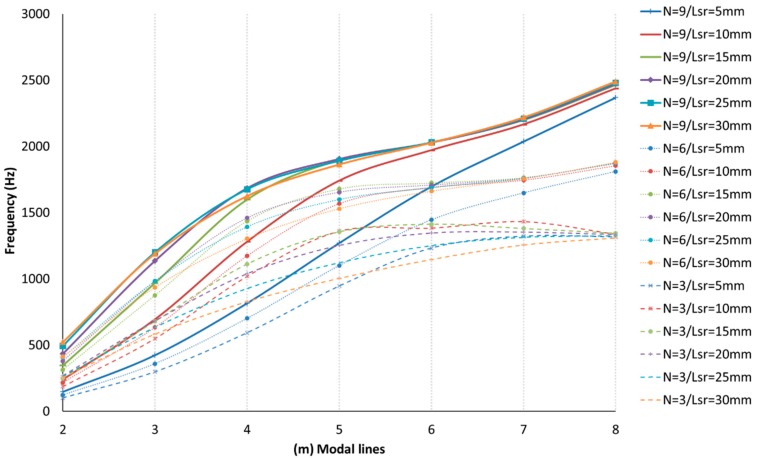
Comparison of drum shell vibration modes (m,0), for 5 > lsr > 30 mm, and three, six, and nine layers of laminate. Equivalent design points are detected, allowing for material savings by maintaining frequency responses for the same vibrational mode.

As shown in Figure 17, different design possibilities are equivalent in frequency just by representing three-layer thickness increases. If we observe the vibration mode m=4, we can obtain the same resonance frequency by using nine layers of laminate and a structural reinforcement of 5 mm or three layers of laminate and a structural reinforcement of 30 mm. This equals a material savings of approximately 67%.

Using the same method, we can obtain similar material savings for the mode m=5, where nine layers of material with a lsr=5 mm or three layers of composite with a lsr=20 mm are used.

Additionally, for the mode m=6, we can save 33% of material and obtain a similar behavior for all values of lsr≥10 mm. For the mode m=4, we find an equivalent resonance in frequency between a laminate of nine layers and lsr=10 mm, and a laminate of six layers and lsr=30 mm, which offers us considerable material savings.

In view of these results, we can affirm that the use of combinations of geometric variables under a modal criterion can offer us design points that allow us to optimize the quantity of material used, lessening production times and the costs associated with production.

## 6. Experimental Validation

**Figure 18:**
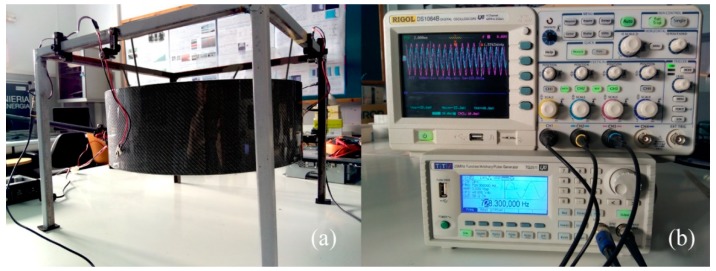
Experimental test. (**a**) The drum shell in free conditions is excited externally to obtain its natural frequencies. (**b**) Wave generator and digital oscilloscope used in this investigation.

In order to experimentally check the effects of the structural reinforcement length, the suitability for the use of this variable, and the results obtained by simulation, an experimental test shown in Figure 18 was carried out, as described in the methods section.

**Figure 19:**
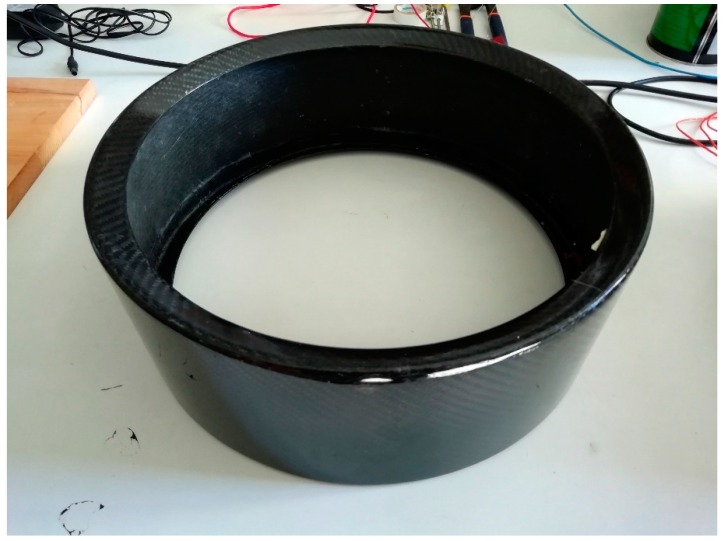
CFRE drum shell made for the experimental validation.

For this purpose, the resonances of the shell made up of three layers of CFRE (N=3, and with a lsr=30 mm) were analyzed. The shell is shown in Figure 19.

As shown in the discussion of the results, this shell allows a material savings of 67% and maintains a value of 828 Hz for the vibration mode (4,0), so it is of great interest for the investigation.

**Figure 20:**
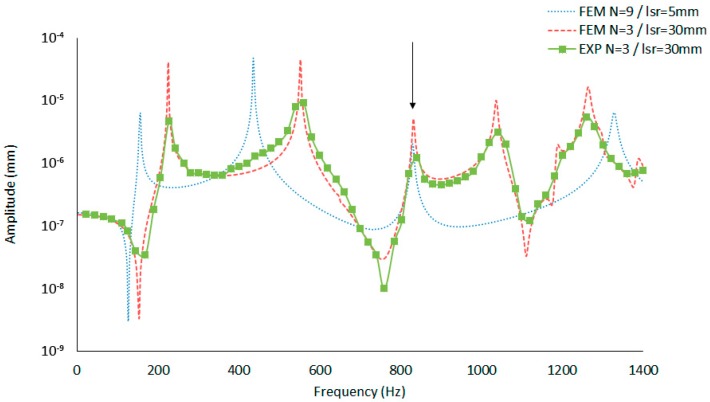
CFRE drum shell amplitude vs. frequency obtained by FEM and experimentally.

As shown by the results in Figure 20, there is a good agreement between the experimental and simulation data.

The vibration mode (4.0) (marked with an arrow) is located at approximately 828 Hz and can be obtained by a different geometric configuration, allowing us to save a lot of material while maintaining the resonances of interest.

## 7. Conclusions

This paper carried out a numerical and experimental study of the possibilities offered using structural features in the manufacture of cylindrical shells for membranophones.

It was demonstrated that the use of geometric variables represents a great potential for acoustic design. Different modal behaviors can be obtained from these geometric variables. Specifically, the combination of those variables in which the general aspect of the product is not modified can allow the desired resonances to be obtained, with great advantages in their manufacture.

The use of existing characteristics in the shells, such as structural reinforcements, has allowed equivalent resonance frequencies to be obtained with very significant material savings of between 33% and 67%, which translates into a reduction in both material costs and production times due to the inherent characteristics of the composite lamination process. The use of fewer layers of laminate avoids material consumption and allows for direct savings in production costs. In addition, as it is a manual process, the time required to generate the laminate by stacking layers is reduced in the same way. The results obtained from this research are applicable to this type of industry and present a low impact, because the process can be carried out without modifying the existing molds and tools. Future work will address new research in this field such as the combination of other materials, the study of industrial processes, and the study of new geometric variables useful for the manufacture of musical instruments.

## Figures and Tables

**Table 1:** Elastic properties of carbon fiber-reinforced epoxy (CFRE) supplied by the manufacturer.

Density	1451 kg·m^−3^
Young Modulus ($E_{1}$)	59.16 × $10^{9}$ Pa
Young Modulus ($E_{2}$)	59.16 × $10^{9}$ Pa
Young Modulus ($E_{3}$)	7.5 × $10^{9}$ Pa
Poisson Ratio ($\upsilon_{12}$)	0.04
Poisson Ratio ($\upsilon_{23}$)	0.3
Poisson Ratio ($\upsilon_{31}$)	0.3
Shear Modulus ($G_{12}$)	17.5 × $10^{9}$ Pa
Shear Modulus ($G_{23}$)	2.7 × $10^{9}$ Pa
Shear Modulus ($G_{31}$)	2.7 × $10^{9}$ Pa
